# Influence of Nanotopography on Early Bone Healing during Controlled Implant Loading

**DOI:** 10.3390/nano10112191

**Published:** 2020-11-03

**Authors:** Renan de Barros e Lima Bueno, Katia J. Ponce, Ana Paula Dias, Dainelys Guadarrama Bello, John B. Brunski, Antonio Nanci

**Affiliations:** 1 Laboratory for the Study of Calcified Tissues and Biomaterials, Department of Stomatology, Faculty of Dental Medicine, Université de Montréal, Montréal, QC H3C3J7, Canada; renanbueno@gmail.com (R.d.B.e.L.B.); kj.ponce@umontreal.ca (K.J.P.); goiab@hotmail.com (A.P.D.); dainelys.guadarrama.bello@umontreal.ca (D.G.B.); 2 Department of Surgery, School of Medicine, Stanford University, Stanford, CA 94305, USA; brunsj6@stanford.edu; 3 Department of Biochemistry and Molecular Medicine, Faculty of Medicine, Université de Montréal, Montréal, QC H3C3J7, Canada

**Keywords:** implant, nanotopography, loading, micromotion, bone healing, histomorphometry, gene expression

## Abstract

Nanoscale surface modifications influence peri-implant cell fate decisions and implant loading generates local tissue deformation, both of which will invariably impact bone healing. The objective of this study is to determine how loading affects healing around implants with nanotopography. Implants with a nanoporous surface were placed in over-sized osteotomies in rat tibiae and held stable by a system that permits controlled loading. Three regimens were applied: (a) no loading, (b) one daily loading session with a force of 1.5N, and (c) two such daily sessions. At 7 days post implantation, animals were sacrificed for histomorphometric and DNA microarray analyses. Implants subjected to no loading or only one daily loading session achieved high bone-implant contact (BIC), bone-implant distance (BID) and bone formation area near the implant (BFAt) values, while those subjected to two daily loading sessions showed less BFAt and BIC and more BID. Gene expression profiles differed between all groups mainly in unidentified genes, and no modulation of genes associated with inflammatory pathways was detected. These results indicate that implants with nanotopography can achieve a high level of bone formation even under micromotion and limit the inflammatory response to the implant surface.

## 1. Introduction

Surface modification strategies at all scales have been proposed for improving the osseointegration of implants, particularly in situations where bone quality is an issue or immediate loading is clinically indicated. Nanoscale features are widely found in nature and provide fascinating physicochemical properties such antibacterial capacity in insects and water repellence in plants [1,2]; they also guide the crosstalk between cells and matrix molecules [3,4,5,6,7]. Various cell types have been shown to respond to nanofeatures and there is now an abundant literature documenting the capacity of nanotopography to influence the activity of osteogenic cells [3,7,8,9], enhance osteogenic differentiation of stem cells and trigger a distinct set of signaling pathways [10,11]. Reports by several groups [12,13,14,15,16,17] have also shown the efficiency of nanoscale surface modification of implants placed in the complex environment of the body. However, these reports dealt with unloaded implants and comparative information on how nanotopographic implants perform under ‘acceptable’ and ‘detrimental’ loading conditions is still lacking.

When implants are placed in function, the imparted forces will cause some degree of micromotion, which generates local strain and stress conditions that deform interfacial tissues, which can influence the bone healing response [18,19].

Yet, few studies have investigated the effect of nanotopography under loading conditions and its impact on the healing events at the bone–implant interface. The objective of this study was to evaluate, using histomorphometry and DNA microarray analysis, the influence of nanotopography on the mechanobiology at the bone–implant interface under well-defined loading conditions. To achieve this, we have taken advantage of a rat tibial loading system that holds the implant stable, thereby allowing the controlled application of forces in a peri-implant environment that sustains de novo bone formation [20]. We have also used a facile oxidative nanopatterning to produce a unique nanoporous surface network that enhances the osteogenic response both in vivo and in vitro [3,17,21]. The results demonstrate that a surface with nanotopography can sustain improved bone formation even under micromotion. A better understanding of the impact of nanoscale surface features on micromotion is expected to foster the creation of implants with rationally engineered surfaces that will prevent detrimental tissue formation while actively promoting bone healing and regeneration around implants.

## 2. Materials and Methods

### 2.1. Surface Modification and Surface Analysis

Commercially pure grade II titanium implants (1.7 mm outer diameter including the threads; Medical Micro Machining Inc, Colfax, CA, United States) were nanotextured using a solution of H_2_SO_4_ and H_2_O_2_ for 2 h; their surface appearance as well as the distribution and size of the nanopores obtained were confirmed using a JEOL JSM-7400F (JEOL Ltd., Tokyo, Japan) field-emission scanning electron microscope (FE-SEM) operated at 1.5 kV, as previously described [3,22,23].

### 2.2. Ethical Approval and Animal Post-Surgical Monitoring

All animal experiments received approval of the Comité de déontologie de l’expérimentation sur les animaux of Université de Montréal (Protocol # 17–113) and are in accordance with the ARRIVE Guidelines for reporting animal research. The behavior and weight of the animals were checked on a daily basis at the University animal facilities throughout the period of experimentation. Moreover, the surgical wound site was inspected for visual signs of inflammation and infection and cleaned every day with a solution of Baxedin^®^ Pre-Op (Omega Laboratories Ltd., Montreal, QC, Canada). The animals were placed in individual cages and allowed to move around freely, and they were given water and food ad libitum.

### 2.3. Surgical Procedure

A detailed description of the implant system and the surgical procedures is given in de Barros et al. [20]. Briefly, 28 male Wistar rats (200–225 g) were anesthetized with Ketamine 100 mg/kg and Xylazine 10 mg/kg. A bone plate was affixed to the superior portion of the tibia using retopins (NTI Kahla GmbH, Kahla, Germany) to guide implant placement, stabilize them and allow controlled loading.

The bony hole into which the surface-modified implants were placed was slightly larger (2.0 mm) than the implant (1.7 mm) in order to create a gap interface [20,24]. After surgery, the rats were given an injection of Buprenorphine (0.05 mg/kg) and Carprofen (0.5 mg/kg). Rats that were used for histomorphometry received an implant on each the tibia, while those for molecular analysis received only one implant on one of the tibiae.

### 2.4. Micromotion System and Loading Regimen

**Figure 1:**
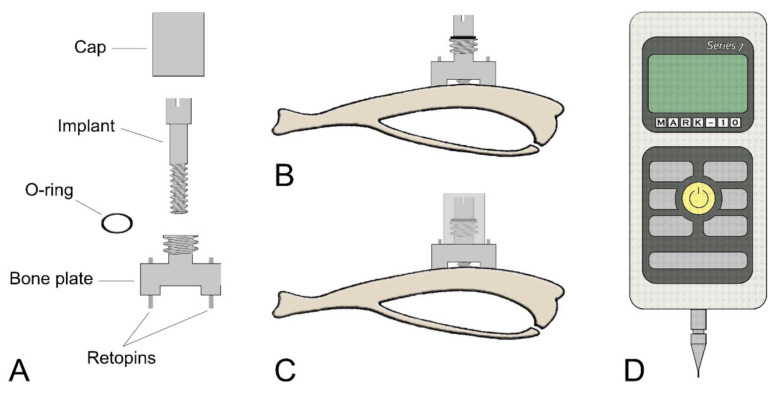
Schematic illustrations of (**A**) each component of the micromotion device and (**B**,**C**) the bone plate positioned on the proximal tibia metaphysis. (**B**) The bone plate is placed using two Ti-alloy Retopins^®^. The implant is guided through the middle of the micromotion device. (**C**) A cap is screwed onto the motion device to hold the implant in place and to secure it against accidental motion due to animal activity. The cap contains a central hole, which allows the loading device to create implant displacement without removing the cap. (**D**) Mark-10 Force Gauge loading component.

A Mark-10 hand-held device force gauge (Copiague, NY, USA) was used for loading implants using a force of 1.5N/cycle following the protocol discussed in Barros et al. [20] (Figure 1). The loading regiments and the experimental groups are summarized in Table 1. During implant loading and daily cleaning of the wound (for both loaded and unloaded groups), the animals were kept under Isoflurane at 1–2% (Baxter, Mississauga, ON, Canada). As discussed in de Barros et al. [20], the Isoflurane anesthesia applied has no significant effect on the bone healing response.

### 2.5. Tissue Processing for Histology

Seven days after implant placement, the animals were anesthetized with a solution of a chloral hydrate (0.4 mg/g) and Xylazine (0.005 mg/g) and sacrificed by an inhalation overdose of Isoflurane. Sample processing for histology/histomorphometry was as described in de Barros et al. [20]. Briefly, tibiae with implants in place were fixed in a mixture of 4% paraformaldehyde and 0.1% glutaraldehyde in 0.1M phosphate buffer, pH 7.2, and decalcified with Planck-Rychlo solution. Following decalcification, the implants were retrieved, and tibiae were processed for embedding in paraffin. All sections were cut longitudinally along the tibiae and stained with hematoxylin and eosin for both histological and histomorphometric analyses. For more detailed histological analyses, some deparaffinized sections were examined using backscatter electron imaging [25] in a JEOL JSM 6460LP variable pressure scanning electron microscope (VP-SEM; JEOL, Tokyo, Japan) operated at 20 kV and 40 Pa.

### 2.6. Histomorphometric Analyses

Only sections that reflected with fidelity the implant outline and showed no interfacial tearing were used for measurements, but in all cases 10 sections per implant were analyzed (sections/implant *n* = 10). Bone-implant contact (BIC), distance between new bone formation and implant surface (bone-implant distance, BID) and bone formation area (BFA) (a) within the osteotomy region (BFAo) and (b) an area corresponding to the region trephined out for microarray analysis (BFAt) were measured. For BID, 30 measurements on each of the 10 sections were taken along the lateral aspects of the implant, for a cumulative number of 300 measurements (*n* = 300) per implant. A detailed description of histomorphometric measurements using the Image-J software (NIH, MD, USA) is given in de Barros et al. [20].

### 2.7. Tissue Processing for RNA Extraction

RNA extraction was carried out as previously reported [20]. Briefly, (1) the implant was removed and immediately placed in 1 mL of TRIzol (Invitrogen, Burlington, ON, Canada), (2) the bone surrounding the implant was trephined under RNAlater (Fisher Scientific, Waltham, MA, USA) irrigation and placed in RNAlater solution (Fisher Scientific) for 48 h and finally placed in the corresponding TRIzol solution used to extract total RNA from any tissue adhering to the implant. The implant- and bone-derived RNA were therefore pooled. Purification and concentration of the total RNA was performed as recommended by manufacturer (Qiagen, Mississauga, ON, Canada). RNA quality was analyzed using the Agilent Bioanalyzer 2100 and only RNA samples with a high integrity number (RNA integrity number (RIN) > 8.5) were used for microarray analyses. The sample size for each group was *n* = 6 (Table 1).

### 2.8. DNA Microarray Design, Hybridization, Data Normalization and Analysis

For DNA microarray analysis, the Gene Chip Rat Gene 2.0 ST Array (Affymetrix, Santa Clara, CA, USA) was used and the samples were examined on a GeneChip^®^ scanner 3000 (Affymetrix, Santa Clara, CA, USA). The Affymetrix^®^ Expression Console™ and Affymetrix Transcriptome Analysis Console (TAC) softwares (Affymetrix, Santa Clara, CA, USA) were used to assess the gene level normalization and gene expression differences respectively. The Ingenuity Pathway Analysis (IPA) software (Qiagen Bioinformatics, Redwood City, CA, USA) was used for the pathway analyses and the gene Ontology (GO) terms were classified according the PANTHER Classification System (http://www.pantherdb.org/). The complete list of the biological process investigated can be found at http://pantherdb.org/panther/prowler.jsp.

### 2.9. Statistical Analyses

For histomorphometry analyses, the data were tested for normal distribution with the Shapiro–Wilk test using the median for each implant (*n* = 5 implants/group). The nonparametric analysis of variance (ANOVA)-type statistic (ATS) was performed [26]. To calculate the ATS, ranks were determined and Proc mixed was used with the ANOVAF option, followed by pairwise comparisons between groups. In all cases, Bonferroni correction was applied. SAS version 9.4 (SAS Institute Inc., Cary, NC, USA) and IBM SPSS Statistics version 25 (IBM Corp., Armonk, NY, USA) were used. Level of power ≥ 80% and *p* value < 0.05 were considered statistically significant.

For microarray, TAC software was used to evaluate the differences in gene expression between the groups by ANOVA, the fold change cut off was set at 2 and the level of significance was 5%.

## 3. Results

### 3.1. Characterization of Surface Topography

**Figure 2:**
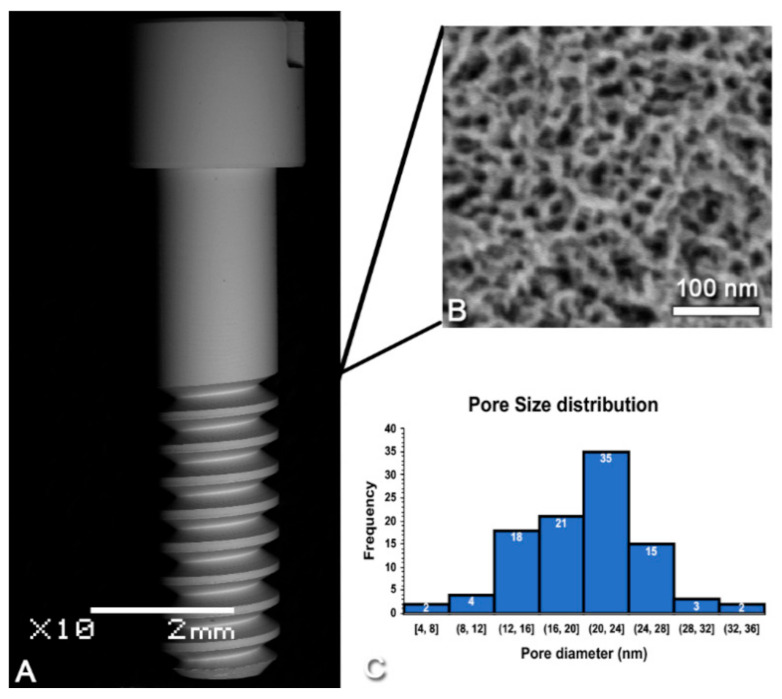
Scanning electron micrographs of (**A**) the screw-shaped implant used and (**B**) the nanoporous topography created by oxidative nanopatterning. (**C**) Size distribution of the nanopores (*n* = 100).

Figure 2A shows an SEM image of the screw-shaped implant used in this study. Consistent with previous reports [3,16,27,28], SEM imaging confirmed the presence of a surface network of nanopores (Figure 2B) with diameters in the range of 20 (±5) nm (Figure 2C), which was homogeneous through the whole implant. The nanopores exhibited depths ranging from 10 to 20 nm values [29].

### 3.2. Post-Surgical Animal Observations

No adverse events (distress, infection and inflammation at the wound site) were detected throughout the 7-day experimental period. The experimental manipulations did not affect the mobility of the animals and, in all groups, weight gain was similar (~57 g/animal).

### 3.3. Histology

**Figure 3:**
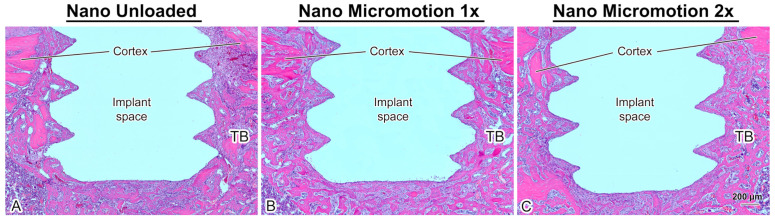
Light microscope images of longitudinally oriented sections stained with hematoxylin and eosin, from (**A**) Nano Unloaded; (**B**) Nano Micromotion 1x and (**C**) Nano Micromotion 2x groups at 7 days post-surgery. New bone forms around the implants in all groups, including between the implant threads. However, signs of disruption of bone healing at the bone–implant interface were noticed in the Nano Micromotion 2x group. TB = trabecular bone in marrow space.

Histological analysis revealed no major inflammatory infiltrate around the implants. In all cases, there was newly formed trabecular bone in the marrow space surrounding the implants and there was no difference in its overall distribution between groups (Figure 3).

**Figure 4:**
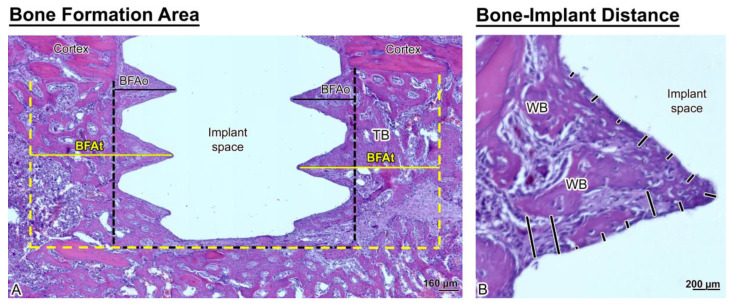
Histological representation of (**A**) bone formation area near the implant (BFAt) (area delimited by the yellow dashed lines) and BFAo (area delimited by the black dashed lines) and (**B**) bone-implant distance (BID) measurements (black lines). Note that the newly formed bone around the implants is trabecular (TB) and woven (WB) in appearance.

**Figure 5:**
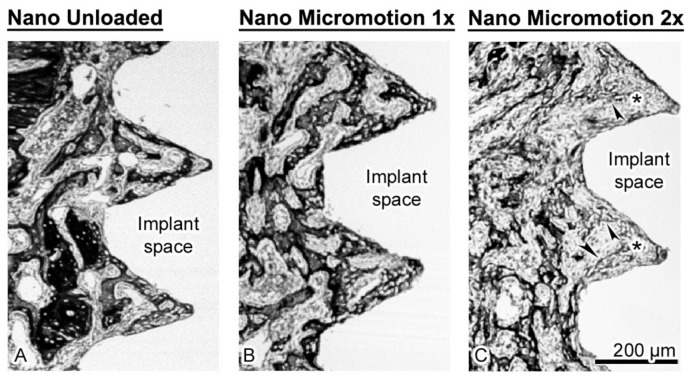
Scanning electron images taken using the backscatter mode showing the (**A**) Nano Unloaded; (**B**) Nano Micromotion 1x and (**C**) Nano Micromotion 2x groups at 7 days post-surgery. In the Nano Unloaded and Nano Micromotion 1x groups, trabecular bone (black) was found all around the implant, including between the threads (**A**,**B**). However, in the Nano Micromotion 2x group the region close to the implants exhibited only sporadic and thinner bone trabeculae (arrowheads) and was mainly occupied by fibrous tissue (asterisks) (**C**).

As illustrated in Figure 4, the trabecular bone is woven in nature (Figure 4B). However, bone formation appeared disrupted close the implant surface in the Nano Micromotion 2x group (Figure 3C and Figure 5C). Backscatter electron imaging allowed us to better visualize the region of disruption between the forming new bone and the implant surface, which was mainly occupied by fibrous-like tissue (Figure 5C).

The few trabeculae found in this region were thinner and emitted a lower backscatter signal, suggesting their organization was less compact. As usual, some osteoclasts (data not shown) were present at the surgical site; however, in all cases there was no notable accumulation of these resorptive cells in proximity to the implant surface.

### 3.4. Histomorphometric Analyses

**Figure 6:**
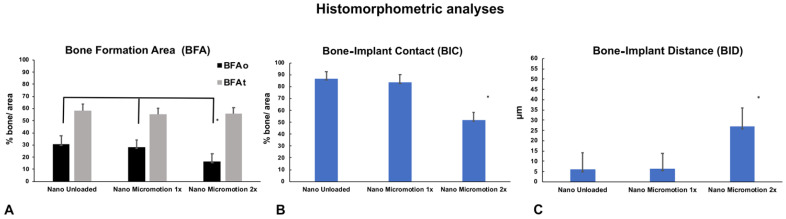
Histomorphometric analyses of bone formation in Nano Unloaded, Nano Micromotion 1x and Nano Micromotion 2x groups at 7 days post-surgery. The Nano Micromotion 2x group showed (**A**) overall lower BFAo; (**B**) lower bone-implant contact (BIC), and (**C**) larger BID compared to the other two groups. Asterisks indicate statistically significant differences.

Histomorphometric analyses corroborated the histological findings. There was no significant difference in bone formation area (BFAt) (Figure 6A and Table 2). However, in the implant osteotomy area (BFAo), bone formation in the Nano Micromotion 2x group showed lower percentages when compared with the other groups (Figure 6A and Table 2). The Nano Micromotion 2x group also showed a lower percentage for BIC (Figure 6B, Table 2) and larger BID when compared with the other two groups (Figure 6C and Table 2). There was no significant difference in all the analyses between the Micromotion 1x and the Unloaded control group (Figure 6A–C and Table 2). The power of the study was 100%.

### 3.5. Gene Expression Profile

Microarray analysis revealed different gene expression profiles between the groups at day 7 post-surgery (Table 3).

**Figure 7:**
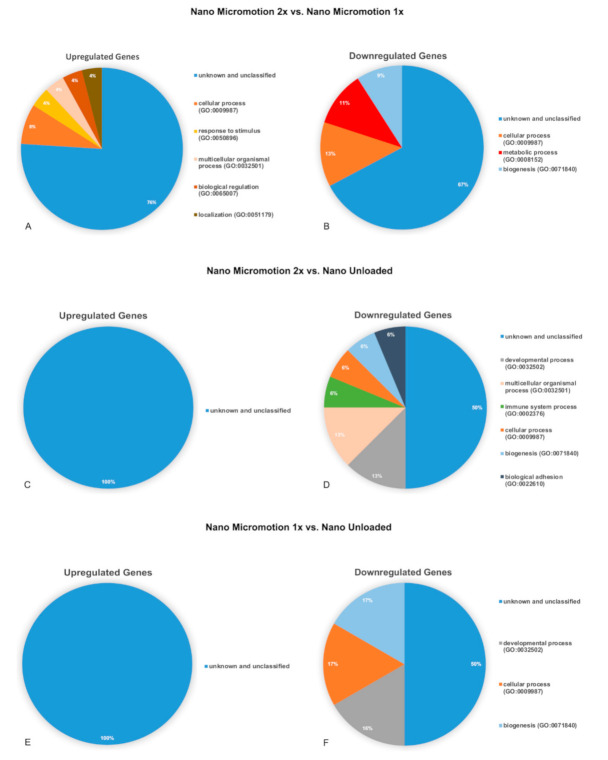
Pie charts showing the distribution of biological process ontologies (BP) related to upregulated (**A**,**C**,**E**) and downregulated (**B**,**D**,**F**) differentially expressed genes (*p* < 0.05). (**A**,**B**) Nano Micromotion 2x group vs. Nano Micromotion 1x group; (**C**,**D**) Nano Micromotion 2x group vs. Nano Unloaded group, and (**E**,**F**) Nano Micromotion 1x group vs. Nano Unloaded group at day 7 post-surgery.

A complete list of differentially expressed genes can be found in Tables S1–S3. The identified genes belonged mainly to the unidentified gene category (Table 3 and Figure 7). The genes were classified into biological processes (BP) according the Panther System Classification in order to evaluate their functional significance. The pie charts show the proportional distribution of up- (Figure 7A,C,E) and downregulated processes (Figure 7B,D,F) at 7 days post-surgery.

The results of the signaling pathways related with bone healing are presented in Tables S4–S6. Although few genes were implicated, the following pathways emerged: Hedgehog, signaling, mRNA processing, endochondral ossification, senescence and autophagy.

## 4. Discussion

The ultimate objective of our work is to better understand the impact of nanoscale surface modifications on micromotion-induced tissue deformation and damage at the tissue–implant interface where cell fate decisions are made. Both loading intensity and frequency play a critical role during bone healing [30,31]. At a high range of loading and micromotion, they can have deleterious outcomes such as the formation of interfacial fibrous tissue and/or interfere with bone repair [31,32,33]. In previous studies using the herein loading system in mice and in rats [20,24], we have demonstrated that multiple daily loading sessions create interfacial stress and strain conditions around machined-surface, screw-shaped implants that can significantly disrupt bone healing and cause fibrous tissue formation. In the present study using similar screw-shaped implants with nanotopography, we also show that simply doubling the number of loading sessions induces major changes at the bone–implant interface but to a lesser degree than similar implants with a machined surface (see below).

The surface generated by the treatment of titanium discs of the same commercial grade II as the implants used herein has been extensively characterized, including roughness values [11,27,29]. The pore size on the screw-shaped implants matches the value (~20 nm) obtained on discs. It should be noted here that the oxidative nanopatterning treatment applied does not cause any micro-roughness under the conditions used in this study [27] and in fact, at 1.5 h of treatment a mono-planar nanoporous surface is achieved [3]. Furthermore, Karazisis et al. [34] demonstrated using screw-shaped titanium implants that the influence of nanotopography on the early biological events of osseointegration is “independent of the underlying microscale topography”. Altogether, this suggests that nanotopography is responsible for the outcomes discussed below.

The BFAo, the BIC, and the BID were affected only in the Nano Micromotion 2x group. A possible explanation is that, compared to the Micromotion 1x group [20], the two loading cycles cause damage in the high-strain regions, which might not be repaired in time before the next loading session [20]. Since the loading is repeated for 7 days, the accumulated damage ultimately leads to an interference in bone healing along the implant surface. In contrast, the BFAt in all groups showed similar values and the placement of the implant into the marrow did not affect bone formation within the broad volume. This could be explained by two factors: (1) the drilling activated the bone modeling sequence in the bone marrow and this process is independent of the implant [35] and/or (2) the stress/strains generated by micromotion have a limited extension from the implant surface.

All groups of implants with nanotopography showed substantial improvement in bone healing when compared to results from a study using equivalent groups of machined surface titanium implants having the same shape and size, placed in the same anatomical site, and loaded using the exact same loading protocol [20]. Comparison with histomorphometry values reported in that study shows that BIC was increased by 1.5 times in the Nano Unloaded group, 1.8 times in the Nano Micromotion 1x and 2.6 times in the Nano Micromotion 2x. This improvement is particularly remarkable in the case of the Micromotion 2x treatment that generates an excessive loading situation that leads to an accumulation of tissue damage during the 7 days of double loading sessions [20]. The increase in BIC is also accompanied by an almost 40% decrease in average BID (Table S7) [20]. While nanotopography does not eliminate interfacial tissue damage, these results suggest that the nanoporous surface has the ability to significantly reduce it. This outcome is consistent with its demonstrated capacity of this nanostructured titanium surface to promote osteogenic activity and also to limit the growth of fibroblastic cells in vitro [11].

The above improvement could also be explained by differences in cell-surface interactions and resulting cell behavior at various scales. We have shown that macroscale surface features can have a detrimental effect on contact osteogenesis when the displacement of the implant relative to bone (micromotion) generates >30% principle strain levels [24,36,37]. If this same micromotion was applied to an implant with nanoscale topography, the displacement of nanoscale features relative to the large surface of the cell might not sufficiently deform entire cells to damage them. Instead, the multiple, small local strains may actually sum up to stimulate cell activity, in this case osteogenesis. Another non-exclusive explanation could be that the biomechanical relationship of cells with the nanoporous implant surface is different. We have recently shown in vitro that the nanoporous surface used here induces the formation of more mature focal adhesions and of numerous filopodia with very fine lateral protrusions [3]. Altogether, these adhesive structures could strengthen the adhesive interaction of cells with the surface and ‘stiffen’ the cells, allowing them to sustain more aggressive strain and stress levels [3,38,39].

The microarray analysis also detected bone-related genes, but these were not differentially expressed. This is consistent with the histological appearance and BFAt values indicating that, in all cases, bone formation was well advanced. Altogether, this suggests that the force applied and resulting implant micromotion were not sufficient to alter the overall osteogenesis during the 7-day interval tested. This may, however, not be the case at earlier time intervals and, as indicated by the BID values, in the Nano Micromotion 2x, also along the implant surface. Analyses at shorter intervals (e.g., 3- and 5-days post-surgery) and narrowing the sampling volume around the implant may put in evidence differentials in gene expression. Laser micro dissection might even allow one to observe local differences in gene expression that are expected at sites of low and high stain/stress points along the implant. Such detailed information would provide a better understanding of how nanotopography improves bone healing, both under stable and loading conditions.

The majority of the genes that were up or downregulated during loading are not classified. This indicates that a number of unsuspected ‘players’ could be involved in implant osseointegration, and these may represent potential targets for promoting bone formation around implants. It is interesting to note that in the report with machined-surface implants [20], micromotion elicited important changes in genes related to inflammatory pathways. With nanoporosity, these pathways were not differentially solicited (Table S8), a finding that is consistent with the lesser inflammatory propensity of the nanoporous surface [22] and that may in part contribute to the overall improvement in bone healing under all loading conditions tested. Finally, nanotopography modulates the expression of some miRNAs during the bone healing [40], such as miR1224, which is implicated in osteolytic bone metastasis [41], and miR140, a regulator of osteogenesis and chondrogenesis [42,43] (Table S9).

## 5. Conclusions

While nanotopography has been shown to stimulate bone formation under stable implant conditions, we show here for the first time that this capacity carries over with loading, at least during initial bone formation. The majority of the genes that were up and downregulated had no classification hit, and genes belonging to inflammatory pathways were not differentially expressed. Compared to machined surface implants tested under similar conditions, the improvements in BIC and BID values in the Nano Micromotion 2x group indicate that the nanoporous surface used alleviates to some degree the consequences of excessive micromotion. This novel finding raises the possibility that implants with nanostructured surfaces could better sustain challenging loading conditions during initial bone healing. It will be interesting to determine whether these gains translate over time during continued loading or secondary implant stabilization.

## Figures and Tables

**Table 1:** Experimental groups and loading regimen.

Group		Number of Implants ^1^	
		Histological-Histomorphometric Analysis ^2^	Microarray Analysis ^3^
1	Nano Unloaded-No Loading	5 implants	6 implants
2	Nano Micromotion 1x-60 cycles/1x-day, 7 days	5 implants	6 implants
3	Nano Micromotion 2x-60 cycles/2x-day, 7 days	5 implants	6 implants

^1^ 28 animals were used for the full experiment. ^2^ 10 animals were used for the histologic-histomorphometric analysis, and implants were placed bilaterally. As discussed in Section 2.6 of the Materials and Methods, from a total of 20 implants placed only 15 (5 implants per group) were used. ^3^ For the microarray analysis, 18 animals (6 implants per group) were used.

**Table 2:** *p* values of analyses. Bonferroni correction was applied for pairwise comparisons.

	Test	Overall *p*-Value	Nano Unloaded vs Nano Micromotion 1x	Nano Unloaded vs Nano Micromotion 2x	Nano Micromotion 1x vs Nano Micromotion 2x
**BFAt**	ANOVA-type statistic	0.4399	1.0000	0.7539	1.0000
**BFAo**	ANOVA-type statistic	0.0012	0.6900	0.0054	0.0075
**BIC**	ANOVA-type statistic	0.0008	0.5244	0.0042	0.0051
**BID**	ANOVA-type statistic	0.0021	0.4050	0.0075	0.0009

**Table 3:** Summary of microarray analysis.

Summary of Microarray Analysis							
Groups	Total Number Differentially Expressed Genes	Total Number Upregulated Genes	Upregulated Unknown and Unclassified Genes	Number Upregulated Genes	Total Number Downregulated Genes	Downregulated Unknown and Unclassified Genes	Number Downregulated Genes
**Nano Micromotion 2x vs. Nano Micromotion 1x:**	69	23	19	4	46	41	5
**Nano Micromotion 2x vs. Nano Unloaded**	29	15	15	0	14	8	6
**Nano Micromotion 1x vs. Nano Unloaded**	14	8	8	0	6	3	3

## Supplementary Materials

The following are available online at https://www.mdpi.com/2079-4991/10/11/2191/s1, Table S1: Differentially known/classified Genes Expressed in the Nano Micromotion 2x group in comparison to the Nano Micromotion 1x group at Day 7, Table S2: Differentially known/classified Genes in the Nano Micromotion 2x group in comparison to the Nano Unloaded group at Day 7, Table S3: Differentially known/classified Genes in the Nano Micromotion 1x group in comparison to the Nano Unloaded group at Day 7, Table S4: List of local pathways obtained for genes differentially expressed in the Nano Micromotion 2x group in comparison to the Nano Micromotion 1x group at day 7, Table S5: List of local pathways obtained for genes differentially expressed in the Nano Micromotion 2x group in comparison to the Nano Unloaded group at day 7 and Table S6: List of local pathways obtained for genes differentially expressed in Nano Micromotion 1x group in comparison to the Nano Unloaded group at day 7. Table S7: Comparison of histomorphometric analyses between machined-surface implants (data from de Barros et al. [20]) vs implants with nanoporous surface. Table S8: List of inflammatory pathways elicited in Machined Surfaces in comparison to the Nanotexture Surfaces at day 7. Table S9: Differentially miRNA expressed genes in the nanotextured surfaces in comparison to the machined surfaces at Day 7.
