# Supplementary material for: Influence of Nanotopography on Early Bone Healing during Controlled Implant Loading
Source: Nanomaterials (Basel). 2020 Nov 3;10(11):2191. doi: 10.3390/nano10112191 (PMC7693286; doi:10.3390/nano10112191)
Supplement: Supplementary file 1 [file nanomaterials-10-02191-s001.pdf]

nanomaterials

MDPI

Supporting Information

Influence of Nanotopography on Early Bone Healing during Controlled Implant Loading

Renan de Barros e Lima Bueno [1], Katia J. Ponce [1], Ana Paula Dias [1], Dainelys Guadarrama Bello [1], John B. Brunski [2] and Antonio Nanci [1,3,*]

[1] Laboratory for the Study of Calcified Tissues and Biomaterials, Department of Stomatology, Faculty of Dental Medicine, Université de Montréal, Montréal, QC H3C3J7, Canada; renanbueno@gmail.com (R.d.B.e.L.B.); kj.ponce@umontreal.ca (K.J.P.); goiab@hotmail.com (A.P.D.); dainelys.guadarrama.bello@umontreal.ca (D.G.B.)

[2] Department of Surgery, School of Medicine, Stanford University, Stanford, CA 94305, USA; brunsj6@stanford.edu

[3] Department of Biochemistry and Molecular Medicine, Faculty of Medicine, Université de Montréal, Montréal, QC H3C3J7, Canada

* Correspondence: antonio.nanci@umontreal.ca; Tel.: +1514-343-5846

In case of Nano Micromotion 2x vs Nano Micromotion 1x, all of the upregulated genes were part of transporter (PC00227) protein classes. Most for downregulated genes were part of the nucleic acid binding (PC00171), signaling molecule (PC00207), transcription factor (PC00218) protein classes (Table S1).

Table S1. Differentially known/ classified Genes Expressed in the Nano Micromotion 2x group in comparison to the Nano Micromotion 1x group at Day 7.

Gene Name	Gene Symbol	Fold Change	p-value
**Friend virus susceptibility 1**	Fv1	2.62	0.01
**olfactory receptor 101**	Olr101	2.22	0.00
**olfactory receptor 522**	Olr522	2.64	0.00
**solute carrier family 38, member 6**	Slc38a6	2.02	0.04
**brain expressed, X-linked 4**	Bex4	-2.04	0.02
**NADH dehydrogenase (ubiquinone) complex I, assembly factor 6**	Ndufaf6	-2.09	0.01
**Sin3-associated polypeptide 18**	Sap18	-2.12	0.02
**small nuclear ribonucleoprotein polypeptide E pseudogene 2 [Source:RGD Symbol;Acc:2318883]**	Snrpep2	-2.21	0.00
**histone cluster 3, H2ba**	Hist3h2ba	-2.55	0.04

In case of Nano Micromotion 2x vs Nano Unloaded, all the upregulated genes didn't hit the protein classification. All the downregulated genes were part of extracellular matrix protein (PC00102) protein classes (Table S2).

Table S2. Differentially known/ classified Genes in the Nano Micromotion 2x group in comparison to the Nano Unloaded group at Day 7.

Gene Name	Gene Symbol	Fold Change	p-value
**arylsulfatase family, member I**	Arsi	-2.11	0.02
**G0/G1switch 2**	G0s2	-2.45	0.04
**hyaluronan and proteoglycan link protein 1**	Hapln1	-3.78	0.04
**collagen, type II, alpha 1**	Col2a1	-4.75	0.01

**C-type lectin domain family 3, member A**	Clec3a	-4.96	0.02
**collagen, type X, alpha 1**	Col10a1	-6.38	0.00

In case of Nano Micromotion 1x vs Nano Unloaded gene profiles, all the upregulated and downregulated genes didn't hit the protein classification. (Table S3).

Table S3. Differentially known/ classified Genes in the Nano Micromotion 1x group in comparison to the Nano Unloaded group at Day 7.

Gene Name	Gene Symbol	Fold Change	p-value
**microRNA 193**	Mir193	-2.01	0.01
**collagen, type II, alpha 1**	Col2a1	-2.76	0.02
**collagen, type X, alpha 1**	Col10a1	-3.52	0.01

PATHWAYS

Table S4. List of local pathways obtained for genes differentially expressed in the Nano Micromotion 2x group in comparison to the Nano Micromotion 1x group at day 7.

Pathway	Total	Up	Up List	Down	Down List	p-value
**Hedgehog Signaling Pathway**	1	0		1	Sap18	0.02
**mRNA Processing**	1	0		1	Snrpep2	0.03

Table S5. List of local pathways obtained for genes differentially expressed in the Nano Micromotion 2x group in comparison to the Nano Unloaded group at day 7.

Pathway	Total	Up	Up List	Down	Down List	p-value
**Endochondral Ossification**	1	0		1	Col2a1	0.02

Table S6. List of local pathways obtained for genes differentially expressed in Nano Micromotion 1x group in comparison to the Nano Unloaded group at day 7.

Pathway	Total	Up	Up List	Down	Down List	p-value
**Endochondral Ossification**	1	0		1	Col2a1	0.01
**Senescence and Autophagy**	1	0		1	Col10a1	0.01

Comparative analysis of nanotopographic with machined-surface implants

It should be noted that for both the machined-surface implant study [1] and the present one with implants with a nanostructured surface: (1) the animals were of the same species, weight and purchased from the same supplier, (2) the system used to stabilize and load implants is exactly the same, (3) the person who placed the implants was the same, and (4) the ALL analyses were carried by the same individuals.

The comparative analysis with machined-surface implants from de Barros et al [1] is summarized in the Table S7: (1) the BID decreased 46% in the Nano Unloaded, 55% in the Nano Micromotion 1x and 37% in the Nano Micromotion 2x groups when compared with the values of their respective machined ones, and (2) the BIC was increased by 51% in Nano Unloaded group, 82% in the Nano Micromotion 1x and 157% in the Nano Micromotion 2x.

Comparative analysis of the average BID's in the two Micromotion 2x groups reveals; (a) a statistically significant difference between Nano Micromotion 2x group when compared Micromotion 2x group (p=0.0005) and a marked tendency to small distances on Nano Micromotion 2x was observed.

nanomaterials

Table S7. Comparison of histomorphometric analyses between machined-surface implants (data from de Barros et al [1] vs implants with nanoporous surface.

Groups	Bone Implant Distance (BID)	Bone Implant Contact (BIC)
**Unloaded**[1]	11.2μm (±1.7)	57.2 % (±7.7)
**Nano Unloaded**	6.0 μm (±8.3)	86.6 % (±6.1)
**Micromotion 1x**[1]	14.6 μm (±2.3)	45.7 % (±7.9)
**Nano Micromotion 1x**	6.5 μm (±7.4)	83.5 % (±6.6)
**Micromotion 2x**[1]	42.5 μm (±3.7)	20.1 % (±6.2)
**Nano Micromotion 2x**	26.9 μm (±8.2)	51.8 % (±6.6)

[1]de Barros, E.L.B.R.; Dias, A.P.; Ponce, K.J.; Wazen, R.; Brunski, J.B.; Nanci, A. Bone healing response in cyclically loaded implants: Comparing zero, one, and two loading sessions per day. J Mech Behav Biomed Mater 2018, 85, 152-161.

Table S8. List of inflammatory pathways elicited in Machined Surfaces in comparison to the Nanotexture Surfaces at day 7.

Pathways Elicited	Machined Implants	Nanotextured Implants
**Cytokines and Inflammatory Response (BioCarta)**	✓	X
**Prostaglandin Synthesis and Regulation**	✓	X
**Type II interferon signaling (IFNG)**	✓	X
**IL-1 Signaling Pathway**	✓	X
**Cellular Compromise, Inflammatory Response**	✓	X

Distinctly from machined surfaces, the nanoporous surfaces upregulated important miRNAs such as miR1224, miR140, Mir3581, Mirlet7f-1 and Mir451a (Table S8).

Table S9. Differentially miRNA expressed genes in the nanotextured surfaces in comparison to the machined surfaces at Day 7.

Groups Comparisons	Gene Name	Fold Change
**Nano Unloaded vs Micromotion 2x**	Mir3581	2.1
**Nano Micromotion 1x vs Unloaded**	Mirlet7f-1/ Mir1224	3.3/ 3.1
**Nano Micromotion 1x vs Micromotion 1x**	Mir- 451a	2.1
**Nano Micromotion 2x vs Unloaded**	Mir1224	3.8

References

1. de Barros, E.L.B.R.; Dias, A.P.; Ponce, K.J.; Wazen, R.; Brunski, J.B.; Nanci, A. Bone healing response in cyclically loaded implants: Comparing zero, one, and two loading sessions per day. J Mech Behav Biomed Mater **2018**, *85*, 152–161.
